# Reply to “Isolating Viable Ancient Bacteria: What You Put In Is What You Get Out”

**DOI:** 10.1128/genomeA.00734-16

**Published:** 2016-08-25

**Authors:** A. Goncharov, S. Grigorjev, A. Karaseva, V. Kolodzhieva, D. Azarov, Y. Akhremenko, L. Tarasova, A. Tikhonov, A. Masharskiy, L. Zueva, A. Suvorov

**Affiliations:** a FSBSI Institute of Experimental Medicine, Saint Petersburg, Russia; b North-West State Medical University named after I. I. Mechnikov, Saint Petersburg, Russia; c Saint Petersburg State University, Saint Petersburg, Russia; d Zoological Institute of the Russian Academy of Sciences, Saint Petersburg, Russia; e M. K. Ammosov North-Eastern Federal University, Yakutsk, Russia

## REPLY

Investigation and description of viable bacteria isolated from ancient permafrost are an essential part of modern paleomicrobiology (1, 2), despite the difficulties with evidence of autochthony of isolates.

In our work, we have described the draft genome of an unusual strain of *Enterococcus faecium* isolated from a paleontological specimen (3). The possibility of contamination with modern microbiota, of course, was considered by us. In this regard, we have used the term “putative ancient” for determination of the isolates.

However, we cannot agree with the opinion that multilocus sequence typing (MLST) allelic profile investigation is sufficient for the estimation of absolute age of bacterial strains, as suggested by Eisenhofer et al. (4). It is clear that housekeeping genes coding for proteins with important functions for bacterial survival are considered for use in MLST schemes because they are stable with respect to rapid genetic modifications. Regarding *Enterococcus faecium*, it has been assumed by Galloway-Peña et al. (5) that divergence of this species into the hospital and commensal phylogenetic clusters occurred no earlier than 300,000 years ago. However, nucleotide differences between sequence types representing the hospital and commensal clusters are relatively small. For example, we have found by MLST profile comparison between ST17 (belonging to the hospital clade) and ST32 (commensal clade) that four of the seven MLST genes are 100% intact and the other three have only 11 single nucleotide polymorphisms (SNPs) in total (95, C → T; 128, C → T; 314, T → C; and 542, T → G in the internal fragment of *atp*A; 171, T → G; 204, G → A; 327, T → C; 348, C → T; 387, C → A; and 435, G → A in *ddl*; and 115, C → T in *purK*). This fact demonstrates that the molecular clock for different housekeeping genes artificially chosen for MLST analysis might go with different speeds depending on the evolutional pressure on the bacteria. As we believe that the rate of molecular evolution is low, we do not expect significant accumulation of mutations in these genes over the last 28,500 years. Besides, the isolation of ST32 among taxonomically distant species of animals and the wide distribution of ST32 in geographically distant regions may indicate that this sequence type is relatively ancient.

Irrespective of the age of the *E. faecium* 58m strain, the presence of genetic regions in its genome that do not have any significant similarity with the genomes of modern prokaryotes (for example, genomic regions from positions 13577 to 23686 in contig 46 and positions 22200 to 23200 in contig 43) is of considerable interest for further study.
